# Eco-Friendly Approaches in Oncology: Developing Holmium (^166^Ho) Glass Microspheres for Hepatocellular Radioembolization

**DOI:** 10.1021/acsomega.4c08734

**Published:** 2025-05-14

**Authors:** Sara Rhaissa Rezende dos Reis, Natália Cristina Gomes-da-Silva, Luciana Magalhães Rebelo Alencar, Alan Silva de Menezes, Frederico Duarte de Menezes, Kirill S. Golokhvast, Eduardo Ricci-Junior, Ralph Santos-Oliveira

**Affiliations:** † Nuclear Engineering Institute, Laboratory of Nanoradiopharmacy and Synthesis of New Radiopharmaceuticals, Brazilian Nuclear Energy Commission, Rio de Janeiro, Rio de Janeiro 21941906, Brazil; ‡ Department of Physics Biophysics and Nanosystems Laboratory, 37892 Federal University of Maranhão, São Luis, Maranhão, 65065690, Brazil; § Federal Institute of Technology of Pernambuco, 119514 DACI/IFPE, Recife, Pernambuco 50740545, Brazil; ∥ Advanced Engineering School (Agrobiotek), Tomsk State University, Lenin Avenue, 36, Tomsk, 634050, Russia; ⊥ Siberian Federal Scientific Center of Agrobiotechnology, Krasnoobsk 633501, Russia; # N.I. Vavilov All-Russian Institute of Plant Genetic Resources, Bolshaya Morskaya Street, St. Petersburg 190000, Russia; ¶ School of Pharmacy, Federal University of Rio de Janeiro, Rio de Janeiro, Rio de Janeiro 21941900, Brazil; ∇ Laboratory of Radiopharmacy and Nanoradiopharmaceuticals, Rio de Janeiro State University, Rio de Janeiro, Rio de Janeiro 23070200, Brazil

## Abstract

Using recycled materials is increasingly recognized as a crucial strategy in today’s global context. The production of glass on a global scale, estimated at approximately 209 million tons annually, underscores the urgent necessity to identify alternative applications for this material. In this milieu, the adoption of recycled glass for applications conducive to health emerges as a significant opportunity, offering dual advantages: mitigating the global surplus of glass and enhancing public health outcomes. In the realm of diseases, oncology, and hepatocellular carcinoma stand out due to their extensive costs and detrimental impact on health. Consequently, this research has been directed toward developing, comprehensively characterization, and in vivo assessment of Ho (Ho-166) holmium-166 doped glass microspheres derived from recycled glass. The findings confirmed the successful formation of these microspheres, marked by a high degree of holmium doping. Moreover, the studies revealed a significant accumulation of the microspheres in the liver, alongside a lack of toxicological effects. Collectively, these results strongly support the potential of recycled glass as a valuable resource for fabricating holmium-doped glass microspheres, offering a promising avenue for liver cancer treatment.

## Graphical abstract

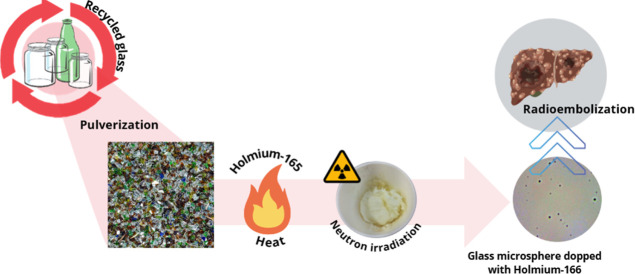

## 1 Introduction

Glass is a material integral to various applications spanning packaging, construction, electronics, and beyond, owing to its versatility, durability, and optical properties. Despite its ubiquitous use and benefits, the lifecycle of glass—from production to disposal—poses several environmental challenges that warrant attention. The production of glass is an energy-intensive process, requiring substantial amounts of heat to melt raw materials like sand (silica), soda ash (sodium carbonate), and limestone (calcium carbonate). This high energy demand, primarily sourced from fossil fuels, contributes significantly to greenhouse gas emissions, exacerbating climate change. Studies estimate that for every ton of glass produced, approximately 682 kg of CO_2_ is emitted, highlighting the carbon-intensive nature of glass manufacturing.^1,2^ Furthermore, the extraction of these raw materials contributes not only to the depletion of natural resources but also has adverse effects on local ecosystems. For instance, silica sand mining can lead to habitat destruction, water pollution, and decreased biodiversity, impacting the ecological balance of affected areas.^3–5^

Beyond production, waste management of glass presents additional environmental concerns. Although glass is inherently recyclable, with the potential for indefinite recycling without losing quality, the reality of glass recycling rates is less than ideal. A significant portion of glass waste is in landfills, which remain indefinitely due to its nonbiodegradable nature. This discrepancy is often due to logistical challenges in the recycling process, contamination of glass with other materials, and a lack of recycling infrastructure in many regions. As a result, the environmental benefits of glass as a recyclable material are not fully realized, contributing to the ongoing waste management issue.^6,7^

Addressing these environmental impacts requires a multifaceted approach. Enhancing the efficiency of glass production processes through technological innovation can reduce energy consumption and emissions. For instance, adopting electric melting technologies or utilizing renewable energy sources can mitigate the carbon footprint of glass manufacturing. Moreover, promoting sustainable mining practices and exploring alternative raw materials can alleviate the ecological strain caused by resource extraction. In terms of waste management, increasing recycling rates is paramount. This necessitates improving recycling infrastructure, raising public awareness about the benefits of glass recycling, and implementing policies that encourage glass recycling.^8^

Glass microspheres represent a pivotal advancement in material science, embodying a fusion of engineering and medical innovation. These minuscule spherical particles, fabricated from silica-based glass, range in diameter from a few micrometers to several hundred micrometers.^9,10^ Their unique physical and chemical properties, such as uniformity in size, inertness, and structural stability, render them invaluable across various applications, from industrial composites and coatings to targeted drug delivery systems.^11–13^

The application of microspheres in medical treatments has been extensively studied, including in rheumatoid arthritis models, such as lithium dysprosium borate microspheres,^14^ as well as in targeted chemoembolization and precision-guided radioembolization. These approaches represent significant advances in cancer therapy, as demonstrated by the use of yttrium-90 (resin and glass) microspheres and poly-l-lactic acid microspheres polymerized with holmium-166, which enhance treatment efficacy while minimizing systemic side effects.^15,16^

The specific properties of different types of microspheres have been well-documented, highlighting their distinct characteristics. Glass microspheres, for instance, are classified as nondegradable and nonporous. Due to their inert nature, they do not induce immunological responses; however, their use in chemoembolization is limited.^15^ In contrast, polymer-based microspheres offer a more versatile alternative, ensuring greater safety for repeated-dose administration. These microspheres can be functionalized for the controlled delivery of antitumor drugs and radioisotopes, enabling more precise and effective release at the target site. However, polymers are more susceptible to neutron irradiation, which can lead to structural degradation, compromising their stability, mechanical integrity, and ultimately, the therapeutic efficacy of the system.^15,17^

Finally, Holmium-166 (Ho-166) microspheres offer distinct advantages over yttrium-90 (Y-90) microspheres for radioembolization, particularly due to their dual emission properties and material characteristics. Unlike Y-90 glass microspheres, which are nondegradable and lack imaging capabilities beyond indirect bremsstrahlung detection, Ho-166 poly­(l-lactic acid) (PLLA) microspheres are biodegradable, allowing for controlled radionuclide release and improved biocompatibility. Their lower density compared to glass microspheres facilitates deeper penetration into tumor vasculature, potentially enhancing therapeutic efficacy. Additionally, the gamma emission of Ho-166 (81 keV, *T*_1_/_2_ = 26.8 h) enables real-time imaging via single-photon emission computed tomography (SPECT), providing superior spatial resolution and direct visualization of microspheres distribution. While the rapid decay of Ho-166’s gamma emission does not reduce radiation exposure relative to Y-90—which lacks gamma emission entirely—the ability to track microspheres in real time is a significant clinical advantage. These features support the potential of Ho-166 microspheres as a more versatile alternative for theranostic applications in radioembolization.

Radioembolization, a form of brachytherapy, leverages the intrinsic characteristics of glass microspheres to deliver targeted radiation therapy.^18^ This minimally invasive procedure is primarily employed in the treatment of certain types of liver cancer, such as hepatocellular carcinoma and metastatic colorectal cancer in the liver.^19^ In this context, the glass microspheres are impregnated with a radioactive isotope emitting beta radiation. These radioactively doped microspheres are then introduced into the hepatic artery through a catheter, transporting them to the liver, where they lodge in the vascular beds, feeding the tumor.^20,21^

The strategic advantage of radioembolization lies in its ability to deliver high doses of localized radiation directly to the tumor site while sparing surrounding healthy tissues.^22^ This targeted approach minimizes systemic side effects and enhances the therapeutic efficacy of radiation. The physical properties of glass microspheres ensure that they remain embolized within the target area, providing a sustained release of radiation over a period typically spanning several days. This controlled release mechanism facilitates a continuous assault on cancer cells, potentially reducing or stabilizing tumor size.^23,24^

Holmium-166 (Ho-166) is a radioactive isotope of the lanthanide element holmium (Ho), which significantly advances radioembolization techniques within nuclear medicine. With a half-life of approximately 26.8 h, ^166^Ho emits both beta radiation, which is effective for therapy, and γ radiation, which allows for imaging and dosimetric evaluation.^17^ Compared to Y-90, which has a longer half-life (64.2 h) and emits higher-energy beta particles (2.28 and 0.94 MeV), Ho-166 offers additional advantages. While Y-90 does not emit γ radiation, only secondary photons or bremsstrahlung,^25^ the dual beta and gamma emissions of Ho-166 provide a significant benefit. Although extending the active lifespan of microspheres is advantageous, the gamma emission of Ho-166 allows for real-time tracking, enhancing the precision and monitoring of microsphere distribution during treatment. This dual-emissive characteristic makes Ho-166 an exceptionally versatile radioisotope for therapeutic and diagnostic purposes in the medical field.

The therapeutic utility of Ho-166 microspheres lies primarily in the treatment of liver malignancies, including both primary liver cancers such as hepatocellular carcinoma (HCC) and liver metastases from other cancers.^26^ The beta radiation emitted by Ho-166 induces cellular damage and death within the tumor, reducing its size or halting its growth. In contrast, γ radiation allows clinicians to visualize the distribution of microspheres within the liver, providing real-time feedback on the treatment’s accuracy and facilitating postprocedural dosimetric assessment. Moreover, the relatively short half-life of Ho-166 contributes to a rapid decline in radiation levels post-treatment, reducing the duration of potential radiation exposure to the patient and medical staff. This aspect, combined with the ability to monitor the treatment process through gamma imaging, enhances the safety and effectiveness of the therapy.^27–29^

In this study, we have produced, fully characterized, and evaluated the use of glass microspheres obtained by recycling glasses dopped with ^166^Ho for hepatocellular therapy.

## 2 Materials and Methods

### 2.1 Reagents

All reagents and solvents used in this study were purchased from Sigma-Aldrich (Brazil).

### 2.2 Pretreatment of the Glass

All glass used in this study was previously washed with a detergent solution and dried at 250 °C for 24 h. The glass used is from a recycling industry in Rio de Janeiro.

### 2.3 Glass Microsphere Dopped with Holmium

The production process is protected by the patent: BR 10 2023 023825-4. Briefly, the recycled glass was used as the primary raw material. This was pulverized using mortar and pestle. Then, a total mass of 20 g of the crushed glass was weighed, added of surfactant and 2 g of Holmium (Holmium chloride hexahydrate) and then were mixed vigorously and heated at 1200 °C for 2 h. After that, the mixture was cooled at room temperature and again pulverized using a mortar and pestle. Then, the powder was washed twice with distilled water and dried at 100 °C for 24 h.

### 2.4 Characterization

#### 2.4.1 Energy Dispersive X-ray Spectroscopy

Scanning electron microscopy (SEM) and energy-dispersive X-ray Spectroscopy (EDS) were employed to obtain the sample’s morphology and composition. Images were obtained with magnifications of up to 15 K times using a SEM (Zeiss, Evo) with a secondary electron detector (SE), while the EDS (Bruker, XFlash 410 M) provided information about the distribution of chemical elements on the surface.

### 2.5 Size Distribution

Microspheres at 10^4^ microspheres/mL concentration were deposited on a glass slide and analyzed in an optical microscope model Binocular Microscope 1600× Olen. The images were analyzed in the ImageJ Software, where the image scales were calibrated with a calibration microscopy slide. The images were then analyzed using the Gwyddion 2.60 software tool to measure distances and directions between points. A total of *n* = 394 particles were calculated.

### 2.6 Formation of Radioactive Holmium (Ho-166) Glass Microspheres by Neutron Irradiation

The methodology used was adopted from Xuan et al.^30^ Briefly, a mass of 1 g of glass microspheres doped with Ho-165 was irradiated at the Argonauta Reactor (Potency of 340 W) installed at the Nuclear Engineering Institute (Brazil). The sample was irradiated for 6 h using a thermal neutron flux of 3.2  ×  10^9^ n cm^–2^ s^–1^ with an average thermal neutron energy of 0.0025 eV. The thermal activation microscopic cross-section was 98.5 ± 0.4 barns.

### 2.7 Radioactivity Measure

The induced activity of the Holmium-166 doped glass microspheres was determined by a gamma spectrometry system with a hyper-purity germanium (HPGe) detector with a diameter of 6.2 cm, 4 cm in height, 41.1 cm^3^ of active volume and 30% detection efficiency; coupled to the multichannel analyzer (Canberra) with 8.192 channels. The detector was surrounded by a lead cover of ∼10 cm to reduce the background. The measurement was standardized at 3600 s (1 h).

### 2.8 Detection Efficiency

The detection efficiency for each energy was determined using a LabSOCS (Laboratory SOurceless Calibration Software, Canberra). For this, it was necessary to design the geometry used in a computational environment by inserting the physical, chemical, and geometric characteristics of the sample holder, the detector, and the sample to be analyzed. After entering the data, the software simulates the detection efficiency values for each energy. Then, the software doubles the number of voxels and repeats the entire process, obeying the convergence criteria and comparing the values until a satisfactory convergence is obtained.

### 2.9 In Vivo Biodistribution: Tissue Deposition

#### 2.9.1 Animals

Experiments were performed on female naive Wistar rats, *n* = 6, weighing 300–350 g. Animals were housed one per cage under controlled conditions of luminosity (12:12 h light and dark cycle) and temperature (21.0 ± 1.0 °C), with free access to water and standard chow. All procedures were approved by the State University of Rio de Janeiro Animal Care and Use Committee (Rio de Janeiro, RJ, Brazil; protocol number CEUA/8059100220/2021), which is consistent with the United States National Institute of Health Guide for Care and Use of Laboratory Animals (National Research Council, 1996).

#### 2.9.2 Animal Preparation

Animals were anesthetized by an intraperitoneal injection (ketamine 100 mg kg^–1^ and xylazine 20 mg kg^–1^)

## 3 Design Protocol

For the biodistribution/tissue deposition studies, 15 μCi/0.1 mL of holmium-166 doped glass microspheres were injected intraperitoneally (i.p.), evaluating the systemic behavior in healthy animals. Animals were sacrificed 24 h postinjection by using an excess of anesthesia (Isoflurane chamber), the blood and organs of interest heart, brain, stomach, intestine, bladder, kidney (right and left), lung (right and left), liver, spleen were immediately dissected out and weighed for quantitative estimation of gamma counts using a gamma counter (Hidex, Turku, Finland). Results were expressed as a percentage of injected dose per organ (% ID/g).

### 3.1 Biochemistry Analysis

Blood samples were collected by cardiac puncture from healthy mice treated (intervention group) with holmium-166 doped glass microspheres at 24 h postintraperitoneal administration (*n* = 3 per group). Then, the blood samples (0.5 mL) were added into microtubes containing 0.5 mL anticoagulant Heparin (Sigma-Aldrich, Brazil). Plasma was separated by centrifugation (5000 rpm, 5 min, 4 °C). The samples were processed according to the manufacturer’s instructions (Bioclin, MG, Brazil) to determine enzymatic activities of alanine aminotransferase (ALT), aspartate aminotransferase (AST), gamma GT (GGT), creatinine (CRE), lactate dehydrogenase pyruvate (LDH-P), glucose (GLU) and amylase (MAS).

### 3.2 Statistical Analyses

In vitro experiments, values are expressed as means ± SD. Differences between groups were tested for significance by one-way ANOVA followed by Tukey multiple comparison tests using GraphPad Prism 8.1 software. A *p*-value of ≤0.05 was considered significant.

## 4 Results

### 4.1 Glass Microsphere Dopped with Holmium

The holmium-166 doped glass microspheres was well formed. Before any processing (as soon as it came out of the oven), a thin, slightly yellowish film was formed (Figure 1A). Then, after pulverization, a yellowish powder is Figure 1B.

**Figure 1:**
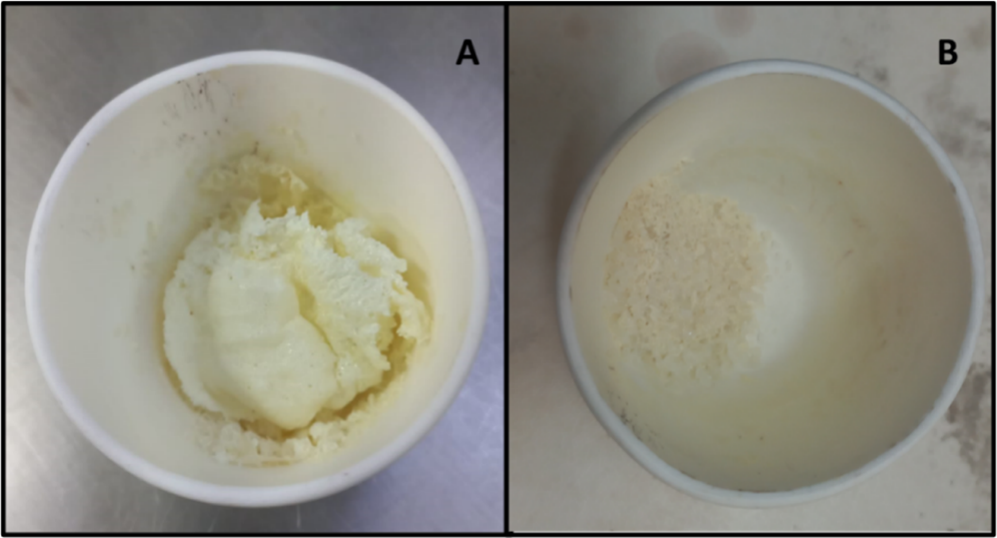
In (A), the glass microsphere is shown to have been dopped with ^165^Ho just after heating. In (B), the glass microsphere was dopped with ^165^Ho after pulverization.

### 4.2 Size Distribution

Figure 2 shows the size distribution of the holmium-165 doped glass microspheres obtained through optical microscopy images. Figure 2A shows the optical microscopy image of the microspheres from the diluted solution (10^4^ microspheres/mL). It is possible to observe the rounded structures of submicrometer size. Figure 2B shows the size distribution statistics of the analyzed microspheres. For *n* = 394 microspheres, a diameter of 1.23 ± 0.65 μm was obtained.

**Figure 2:**
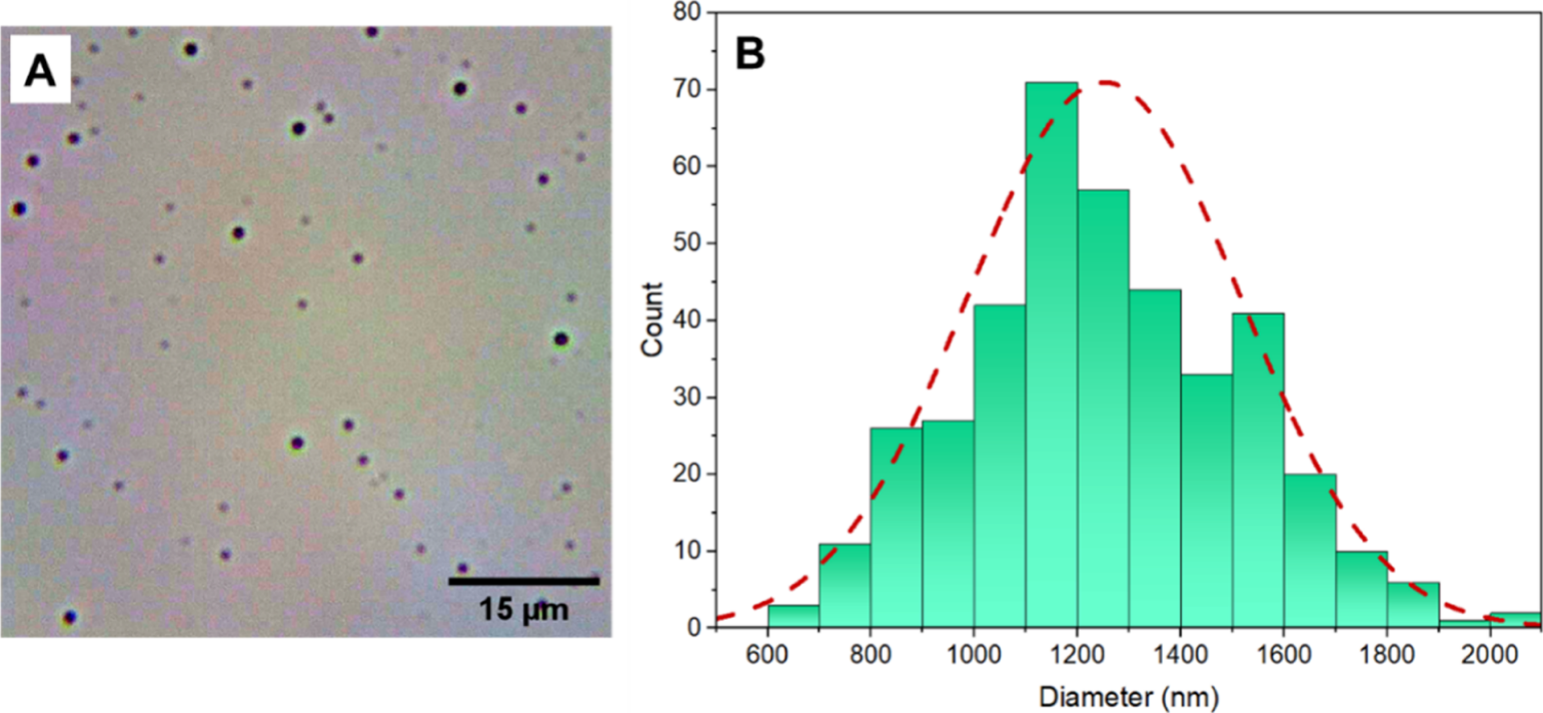
Size distribution. (A). Optical microscopy image of the holmium-165 doped glass microspheres from the diluted solution (10^4^ microspheres/mL). (B). Size distribution statistics of the analyzed microspheres. For *n* = 394 microspheres, a diameter of 1.23 ± 0.65 μm was obtained.

### 4.3 Energy Dispersive X-ray Spectroscopy

Energy-dispersive X-ray spectroscopy (EDS) performed in the holmium-165 doped glass microspheres (Figure 3) demonstrated the presence of 165Ho on the glass microsphere, corroborating the efficacy of the methodology used. It is also possible to observe the presence of aluminum (Al) and silicon (Si), common elements in the composition of glasses.

**Figure 3:**
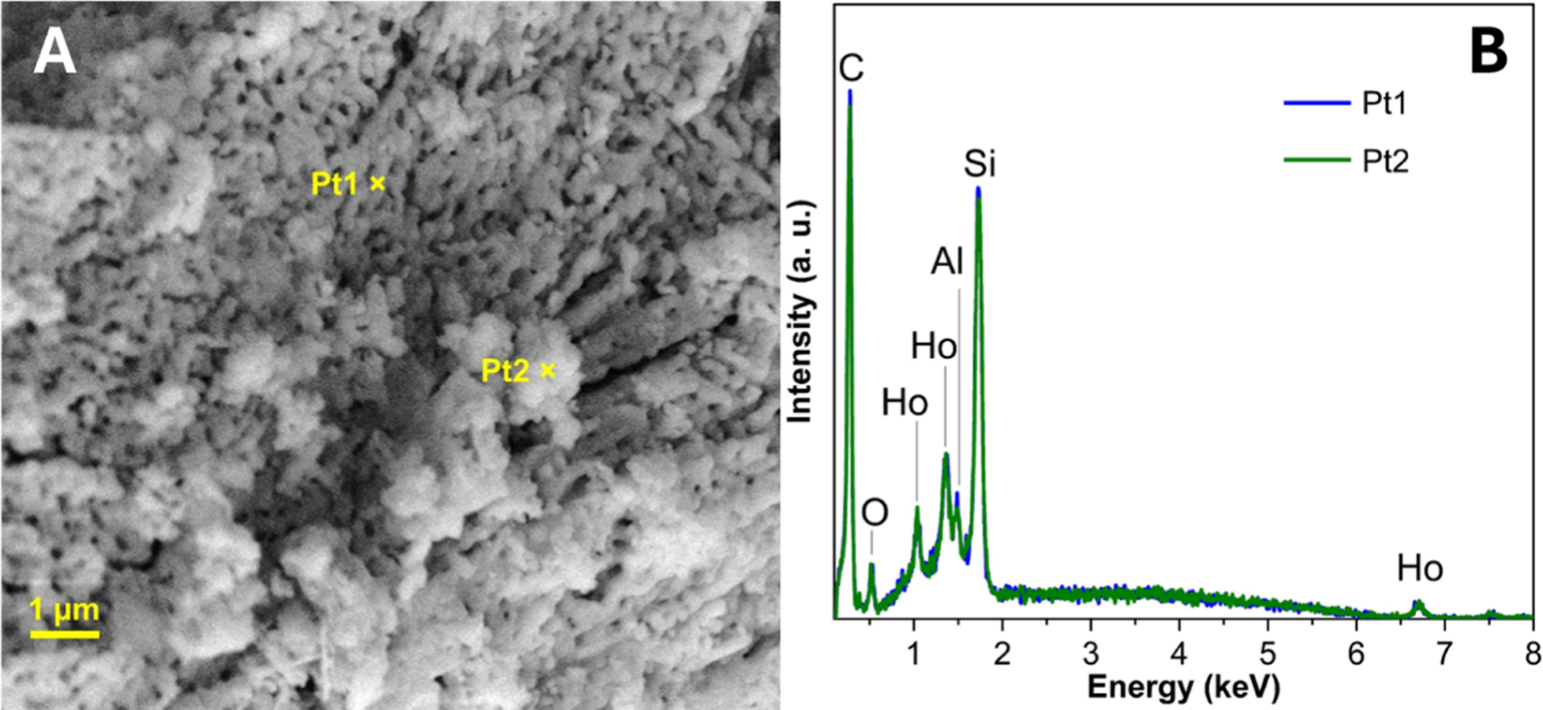
In (A) is an overview image with a magnification of 15.0 K. In (B) is the EDS analysis demonstrating the percentual and the presence of holmium-165 doped glass microspheres. Formation of Radioactive Holmium (^166^Ho) Glass Microspheres by Reactor Irradiation.

The irradiation using a thermal neutron flux of 3.2 × 10^9^ n cm^–2^ s^–1^ with an average thermal neutron energy of 0.0025 eV and a thermal activation microscopic cross-section of 98.5 ± 0.4 barns for 6 h was sufficient to convert Ho-165 into Ho-166.

### 4.4 Radioactivity Measure

The radioactive measurement in the glass microspheres was confirmed by the radioactive measurement (Figure 4).

**Figure 4:**
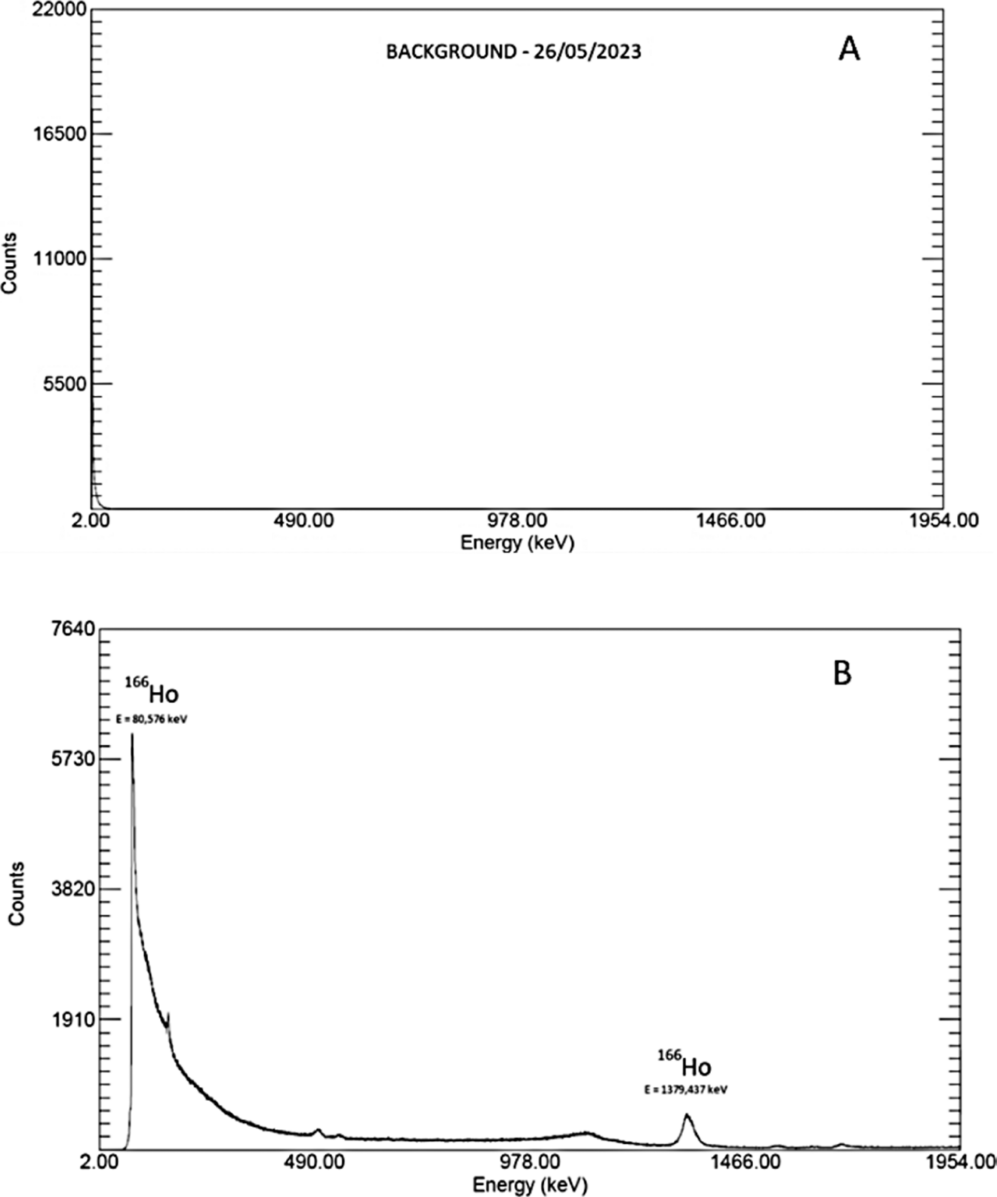
HPGe gamma spectroscopy analysis of Holmium-doped microspheres. (A) Background spectrum recorded on 26/05/2023, showing no significant peaks in the measured energy range. (B) Spectrum of the microspheres after neutron activation, confirming the conversion of stable Ho-165 into radioactive Ho-166 through neutron capture. Characteristic gamma emissions of Ho-166 are observed, including peaks at approximately 80.6 keV and 1379.5 keV, corresponding to its decay.

### 4.5 In Vivo Biodistribution: Tissue Deposition

The Figure 5 expresses the biodistribution of the holmium-166 doped glass microspheres through the percentage of activity applied per gram of each corresponding organ. The highest uptake was observed in the kidneys and bladder (5). In a detailed analysis, removing the organs that showed the most increased uptake revealed a ubiquitous deposition (with low uptake) in several organs.

**Figure 5:**
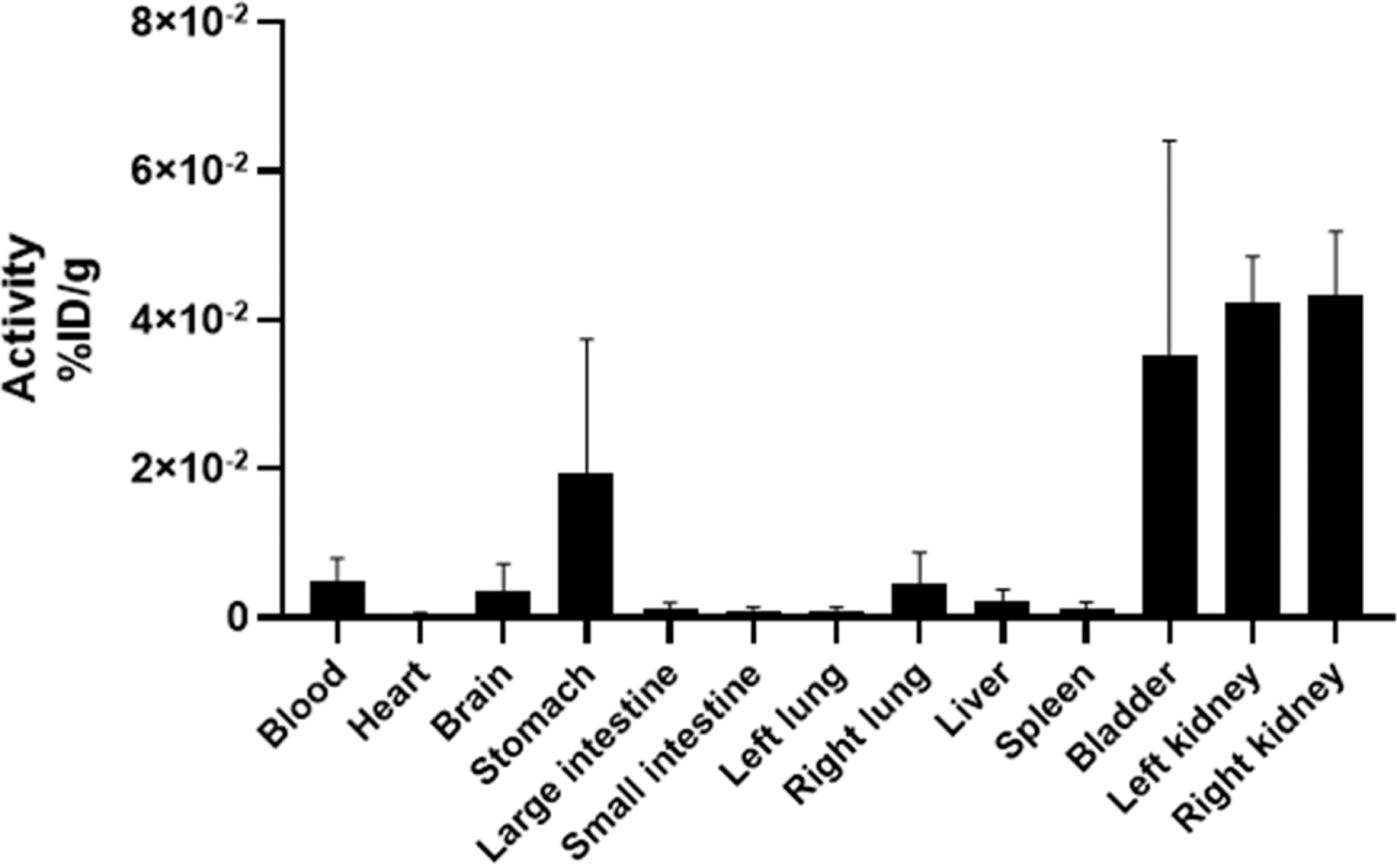
Biodistribution of the holmium-166 doped glass microspheres in healthy rats (*n* = 4). In 5, it is possible to observe a high uptake in clearance organs. The standard deviation of the mean was used.

### 4.6 Biochemistry Analysis

The main biochemical parameters used to assess the potential cytotoxicity of glass microspheres in whole blood from healthy animals are presented in Table 1. For this evaluation, we analyzed key biomarkers, including alanine aminotransferase (ALT), aspartate aminotransferase (AST), gamma-glutamyltransferase (GGT), creatinine (CRE), lactate dehydrogenase (LDH-P), and glucose (GLU).

**Table 1:** Main Biochemical Parameters after Intraperitoneal Injection of Holmium-166 Doped Glass Microspheres in Healthy Female Wistar Rats

parameters (units)	average ± SD	references
ALT (U/L)	34.3	54.3 ± 10.2
AST (U/L)	13.2	80.7 ± 11.7
GGT (U/L)	4.6	4.31 ± 1.5
CRE (mg/dL)	0 ± 0	0.44 ± 0.1
AMS (U/L)	163.8	72 ± 1.5
LDH-P (mg/L)	896.6	724 ± 61.5
GLU (mg/dL)	175.4	91.6 ± 21.15

## 5 Discussion

The micrometric size was evaluated and confirmed by statistical size distribution measurements by light microscopy. The impregnation of the glass microsphere has been confirmed by EDS and HPGe measurements. In the first observation, the presence of Ho-165 was identified, and in the second, the recently converted Ho-165 into Hop-166 after irradiation was noted. It is important to highlight that the HPGe assay revealed the two main peaks of Ho-166: the first (most intense) with an energy of 80.576 keV and the second with an energy of 1379.437 keV. These values align with the literature, particularly with the findings of Bagheri and collaborators.^31^

Additionally, is important to notice that although energy-dispersive X-ray spectroscopy (EDS) confirms the presence of holmium, aluminum, and silicon in the recycled glass used for microsphere production, a comprehensive compositional analysis of the recycled glass remains absent. Given that the material originates from a recycling industry, it is crucial to account for potential contaminants, such as heavy metals, nonglass residues, or organic impurities,^32,33^ which might inadvertently affect the performance or safety of the final product. A detailed compositional analysis could ensure consistency in the microsphere quality and reliability of the radioembolization therapy. Such an evaluation would not only align with stringent material safety protocols but also strengthen the ecological and clinical sustainability of using recycled materials in medical applications. Including this analysis would mitigate variability associated with diverse recycling sources, ensuring reproducible and safe outcomes for clinical use. Although the incorporation of holmium into the glass matrix via high-temperature melting minimizes the likelihood of holmium release, long-term studies are essential to confirm this stability under various conditions, including exposure to bodily fluids. Such analyses would provide critical insights into the durability of the microspheres during storage, handling, and postadministration. Demonstrating the absence of holmium leaching and structural degradation over time would not only bolster confidence in their safety and efficacy but also align with regulatory expectations for materials intended for clinical use.

Clinical considerations regarding the size of the glass microspheres were emerging. Anderson et al.^34^ concluded that the ideal proportions of treatment success were achieved with smaller particles, which impacted the design of the microspheres when considering tumor/hepatic distribution ratios, as their specific diameters allow for better embolization in the terminal arteries of the tumor.^20,35,36^ Meade et al. evaluated the distribution of microspheres of different sizes in experimental liver tumors. They concluded that smaller particles were preferentially lodged in tumors compared to larger particles, which had a distribution in normal parenchyma.^37^ In this direction, the microspheres produced in this study are aligned with the literature for clinical application.

The biodistribution assay indicates the usefulness and effectiveness of holmium-166 doped glass microspheres, allowing us to evaluate and quantify the uptake of the microesphere in tissues and determine the excretion. The data showed that the holmium-166 doped glass microspheres had high renal clearance (Figure 5), with reduced uptake by other organs. It is possible to observe a ubiquitous distribution, but with an irrelevant percentage. The increased uptake of microspheres in the kidneys and bladder may be attributed to their observed diameter (1.23 ± 0.65 μm), as smaller particles are more efficiently cleared through renal excretion.^38^ The low hepatic uptake does not become an impasse in the use of holmium-166 doped glass microspheres, being justified by the hepatic radioembolization technique itself, in which the holmium-166 doped glass microspheres reach the liver tumor tissue by the hepatic arteries, due to hepatic blood flow causing the least possible damage to adjacent healthy tissues.^39,40^

Regarding the safety of the radioembolization using radioactive microspheres (mainly Y-90-glass microspheres) there some studies reporting cases of toxicities related to radioembolization with these microspheres.^41,42^ Our data, as determined by the biochemical analysis, showed that no toxic effect has been observed at the administered dose and the amount of ^166^Ho-glass microsphere used. Is possible to observe that both ALT and AST were not altered, suggesting the absence of acute liver toxicity related to the administration of holmium-166 doped glass microspheres.

It is crucial to acknowledge that the outcomes observed may significantly vary with the radioembolization procedure, given that an extensive quantity of holmium-166 doped glass microspheres will be delivered to the liver. This differential distribution and accumulation of holmium-166 doped glass microspheres are pivotal in understanding the procedural efficacy and potential therapeutic impact on liver tissue, necessitating a tailored approach to evaluate the clinical outcomes associated with this treatment modality.

The simultaneous evaluation of LDH (Lactate Dehydrogenase) and (AMS) amylase levels postradioembolization can provide a more comprehensive understanding of the procedure’s systemic impact. An elevation in LDH in conjunction with changes in amylase levels could indicate not only localized tissue damage within the liver but also potential adverse effects on adjacent organs such as the pancreas. However, due the very low amount of 166Ho-glass microsphere in pancreas the alteration in the LDH and AMS is probably a systemic response due the radiation.^43,44^

While our study provides important insights into the biodistribution of holmium-166 doped glass microspheres, a direct comparison of safety outcomes with clinical radioembolization studies is not appropriate due to differences in administration route and dosage. The intraperitoneal administration used in this work does not fully replicate the intra-arterial delivery of microspheres in clinical radioembolization procedures. However, the findings highlight the potential of our holmium-166 doped glass microspheres, which are smaller than those currently used in clinical practice, for future radioembolization applications. Given the limited data on biodistribution following intraperitoneal administration, further studies are necessary to evaluate safety in a manner that aligns with clinical standards. Future investigations should include dose-escalation studies, intravenous or intra-arterial administration to better simulate clinical conditions, and histopathological assessments to evaluate potential tissue toxicity. These steps will be essential in determining the safety profile and translational potential of Ho-166 microspheres for theranostic applications in cancer treatment.

## 6 Conclusion

The presented data elucidates the feasibility of fabricating medical devices, specifically glass microspheres doped with Ho-166, utilizing recycled glass materials. Despite the preliminary nature of these findings, they robustly support the practicality of this innovative technique. The employment of recycled glass in the production of devices designated for liver cancer treatment through the intricate process of radioembolization underscores a significant advancement in recycling technologies, integrating sustainability into the realm of medical therapeutics.

Furthermore, the research highlights the efficiency and high yield of doping glass microspheres with Holmium. This process facilitates the subsequent conversion of Holmium into its radioactive isotope, Ho-166, which is notable for its effective renal excretion profile. Such a characteristic is paramount for minimizing potential toxicity and enhancing the safety profile of the therapeutic intervention.

In addition, the study provides compelling evidence regarding the safety of the developed device, as demonstrated by the absence of significant biochemical alterations in vivo. This aspect reinforces the device’s potential for clinical application, offering a promising avenue for the treatment of liver cancer with an added benefit of promoting environmental sustainability through the use of recycled materials. Collectively, these insights pave the way for further research and development in the integration of eco-friendly materials in medical device manufacturing, potentially transforming practices in the field with an emphasis on sustainability and safety.

## Data Availability

A patent is filed for that data.
